# Impact of probable bruxism on the oral health-related quality of life among police officers and their relatives

**DOI:** 10.4317/jced.62500

**Published:** 2025-02-01

**Authors:** Jenny Teresa Atuncar-Salazar, Gustavo Augusto Huertas-Mogollón, Evelyn Alvarez Vidigal, Roxana Patricia López-Ramos

**Affiliations:** 1 Postgraduate in Stomatology Universidad Científica del Sur. Lima, Perú; 2 Division of Oral Rehabilitation. Universidad Científica del Sur. Lima, Perú; 3 Division of Paediatric Dentistry. School of Dentistry. Universidad Científica del Sur. Lima, Perú

## Abstract

**Background:**

Bruxism is a global oral disorder that can negatively affect oral health-related quality of life (OHRQoL). Nonetheless, there are few studies regarding police officers. The aim of this study was to determine the impact of probable bruxism on quality of life among police officers and their relatives.

**Material and Methods:**

This cross-sectional study included 243 police officers, and their relatives aged 18-70 years who attended the dental service of a Peruvian National Police Hospital. They were evaluated through a questionnaire of probable bruxism, including a clinical oral examination. The Oral Health Impact Profile (OHIP-14) questionnaire was also used to determine the impact of OHRQoL. Univariate and bivariate statistical analyses were performed. In addition, multivariate analysis was performed via the statistical program STATA version 18.

**Results:**

The mean OHIP-14 score was 13.4 ± 8.0, and 48.6% of the participants had probable bruxism. Statistically significant differences were found in questionnaire domains such as physical pain (*p*=0.001), psychological distress (*p*=0.012), social disability (*p*=0.002) and handicap (*p*=0.007). Multivariate analysis, adjusted for age in years, sex, type of insurance, level of education and degree of dental wear showed the mean OHIP-14 total score was 2.38 points higher in patients with probable bruxism (95% CI; 0.75, 4.71).

**Conclusions:**

Probable bruxism had a negative impact on the oral health-related quality of life of police officers and their relatives.

** Key words:**Bruxism, quality of life, adults.

## Introduction

Bruxism is a tooth grinding habit that occurs both day and night, causing changes in tooth structure, permanent lesions and discomfort, such as jaw pain and headaches (1). Also, bruxism has been defined as a constant movement of the muscles at the level of the jaw in which a clenching of the teeth and/or jaw can be identified (2). The classification of bruxism is divided into three evidence-based categories: ‘possible’ bruxism with patient self-reports through questionnaires and anamnesis; ‘probable’ bruxism with visible clinical signs and self-reports; and ‘definite’ bruxism with self-reports, clinical examinations and polysomnographic recordings, preferably with audio-visual recordings (2,3).

It has been reported that bruxism affects 6% to 91% of the population worldwide (4). Its prevalence varies widely, with waking bruxism affecting 22% to 30% of the adult population, whereas sleep bruxism affects 1% to 15% of the population (1,5). Diverse studies indicate that sleep bruxism could be associated with dental wear and poor oral health-related quality of life (6) or negatively affects quality of life and quality of sleep (7).

It is known that oral problems are responsible for decreasing the quality of life of individuals due to prolonged pain and functional, aesthetic, nutritional, and psychological issues (8). The impact of oral health on quality of life can be measured by an instrument that assesses ‘functional limitation, physical pain, psychological distress, psychological discomfort, handicap, and physical, psychological and social disability (10,11). Moreover, the self-perception of the individual could achieve improvements in treatments for the benefit of their health (12).

According to several studies, bruxism is present in different populations and can cause unfavorable consequences on individuals’ oral and general health and affect negatively the OHRQoL (11). Thus, sleep, daytime, and mixed bruxism were positively correlated with lower OHRQoL (12). Other studies have shown that bruxers had a lower OHRQoL than nonbruxers (13), and medical students with bruxism had negatively affected their OHRQoL (16). Also, Thetakala *et al*. reported that active sleep bruxism had a negative impact on OHRQoL among the inmates of a penal institution (15).

Despite the above mentioned, a systematic review concluded that there is not yet enough scientific evidence to support or refute the association between a type of bruxism and quality of life (16). Besides, police officers are vulnerable individuals due to their work demands which could affects their families. Therefore, the present study aimed to determine the impact of probable bruxism on the oral health related quality of life of police officers and their relatives attended at a Peruvian National Police Hospital.

## Material and Methods

This study was approved by the Ethics Committee of the Universidad Científica del Sur (POS-53-2023-00344) and authorized by the Peruvian National Police Hospital. All participants were informed about the objectives of the study and written informed consents were obtained before the study from all patients.

-Study population and data collection

This observational, descriptive, and cross-sectional study was conducted on police officers and their relatives who attended the dental department of a Peruvian National Police Hospital in Lima, Peru. The sample size was determined using Epidat version 4.2 software, significance level of 0.05 and a power of 0.80. Hence, the study sample was composed of 243 police officers and their relatives aged 18-70 years, with apparent good general health. Individuals with genetic and mental alterations, orthodontic treatment, complete or edentulous prostheses, or treatment for bruxism were excluded.

On the day of the dental appointment, the participants were invited to answer two questionnaires: one related to probable bruxism diagnosis (11) and other related to OHRQoL using the Peruvian version of the OHIP-14 (17). All these data were collected by a single examiner who was a dentist trained in the intonation and reading of questionnaire questions. The clinical assessment of probable bruxism was conducted for the same examiner who has undergone a calibration. In this process, ten individuals were examined by the examiner with an interval of 15 days. This calibration process was conducted by an expert of oral rehabilitation. The kappa value for inter- and intraexaminer reliability were 0.880 and 0.936, respectively, resulting in very good agreement.

-Probable Bruxism’s data

In this study, a questionnaire developed by Dr. Celia Elena del Perpetuo Socorro Mendiburu Zavala based on other authors (11,18-20) was adapted from the Mexican Spanish version (11) to the Peruvian context. This version was pilot tested on a convenience sample of 30 individuals aged 18-70 years who did not participate in the final sample (21). Then, a revision panel consisting of two postgraduate professors in Oral Rehabilitation, and three postgraduate professors in Oral Health areas, all fluent in Spanish, reviewed the results and in consensus developed the Peruvian Spanish version of the questionnaire named probable bruxism instrument (11).

This instrument consists of two sections: 1) Self-report questionnaire which contains 8 self-report questions, as well as anamnesis of symptoms in the last 6 months, and 2) the oral clinical examination which register oral signs of bruxism such as lingual indentations, thickening of the jugal linea alba, gingival recessions, presence of exostosis and/or palatal or lingual torus, hypertrophy of the masseter muscles, xerostomia, fracture of restorations and/or teeth, limitation of mouth opening, tooth mobility without the presence of periodontal disease, presence of tooth wear facets resulting from tooth rubbing or clenching and tooth wear. The Tooth Wear Assessment System was used to determine the presence of tooth wear (TWES 2.0) (22). Hence, this instrument was added to the questionnaire for classifying the severity of the tooth wear.

For data collection, all participants answered the 8 self-report questions. Then, an oral clinical examination was conducted with a mouth mirror in a dental chair. The responses of the questionnaire were dichotomous (yes or no), and the diagnosis of probable bruxism was determined when the participants answered “yes” to questions 1 and/or 2, 7 and/or 8 and when they had 2 or more signs or symptoms (11).

-Individual’s OHRQoL data 

The Peruvian version of the OHIP-14 sp questionnaire for assessing the impact of OHRQoL was used in this study. This instrument consists of 14 questions divided into 7 dimensions: ‘functional limitation, physical pain, psychological discomfort, physical disability, psychological disability, social disability and handicap (10). The higher the score is, the lower the OHRQoL; conversely, the lower the score is, the higher the OHRQoL (17).

On the dental appointment, this questionnaire was applied to the participants by a previously trained examiner. Thus, they answered all the questions in a quiet environment.

-Statistical analysis

Descriptive analysis was performed using absolute and relative frequencies for qualitative variables (bruxism, sex, age, education level, type of insurance and tooth wear) and measures of central tendency and dispersion for quantitative variables (OHRQoL instrument: OHIP-14: total score and score for each dimension). For the bivariate statistical analysis, after determination of normality, the nonparametric Mann‒Whitney U test was applied. Additionally, a linear regression analysis adjusted for age, sex and type of insurance was performed. The significance level was set at *p*<0.05. The statistical software used was STATA version 18 in Spanish.

## Results

The main sociodemographic characteristics of the study population are shown in  Table 1. The sample consisted of 243 participants with a mean age of 54.9 ± 11.8 years. The majority were male (58.4%) and 46.9% had a technical education level. Most of the participants were active police officers (36.6%), and 28.4% were their relatives. Regarding the oral conditions of the participants, a total of 48.6% had probable bruxism while 45.7% had moderate tooth wear. In relation to OHRQoL, the total OHIP-14 mean score was 13.4 ± 8.0.

According to the bivariate analysis of factors associated with probable bruxism, no statistically significant differences were found according to sex, age, type of insurance, education level and degree of tooth wear variables ( Table 2).

Regarding probable bruxism and OHRQoL, statistically significant differences were found in individual domains: physical pain domain (*p*=0.001), psychological discomfort domain (*p*=0.012), social disability domain (*p*=0.002), and handicap domain (*p*=0.007). A higher OHIP-14 mean score was associated with a lower OHRQoL (*p*=0.012) ( Table 3).

The multivariate analysis assessed the relation between OHRQoL with probable bruxism. Thus, it was adjusted with variables: age in years, sex, type of insurance, education level and type of tooth wear. The mean OHIP-14 total score was higher in individuals with probable bruxism than in those without bruxism (95% CI; 0.37, 4.39) ( Table 4).

## Discussion

The purpose of this study was to determine the impact of probable bruxism on the OHRQoL among police officers and their relatives. At this respect, there are crucial factors associated with bruxism that include emotional stress; tobacco, alcohol or coffee consumption; and obstructive sleep apnea and anxiety disorders (23). Also, it is even associated with dental wear (6).

Regarding police officers, emotional stress was associated with bruxism, independently of the type of work, and they are most likely to suffer from tooth wear and sleep disorders (24,25). Thus, these disorders could induce possible negative consequences in the family environment due to their effects on health, sleep and stress.

In this investigation, it was found that police officers and/or their relatives with probable bruxism have lower OHRQoL. These results agree with the findings of other studies regarding probable bruxism conducted by Mendiburu *et al*. (11) in Mexico, Yildirim *et al*. in Turkey (13) and Câmara *et al*. in Brazil (8). Likewise, significant correlations between OHRQoL and probable bruxism have also been reported in diverse studies (7,12,14,15). In respect of our results, it must be state that bruxism negatively affects police officers and their relative’s quality of life. This could be explained due to individuals with oral problems reported to have higher rates of pain and suffering that could cause functional and aesthetic problems with nutritional and psychological consequences (8).

Furthermore, we found differences regarding physical pain domain, psychological distress domain, social disability, and handicap domain. In contrast to Yildirim *et al*., who reported differences in all dimensions of the OHRQoL instrument, indicating that it was negatively affected in patients with probable bruxism (13). Similarly, Câmara *et al*. reported that all instrument dimensions were affected. This could be because they considered four dimensions: oral function, orofacial appearance, orofacial pain and psychosocial impact (7). As a result, probable bruxism was related to physical pain, psychological distress, social disability, and handicap. Hence, this could be explained due to participants had a tendency of psychosocial, emotional and pain that can causes difficulties to concentrate and interfere daily works and activities.

In the present study, there was no association between dental wear and bruxism, which agreed with Bronkhorst *et al*. and Beddis *et al*., who reported that bruxism activity should not be deduced only from the presence of tooth wear (26,27). In this research, the TWES 2.0 tooth wear assessment system (22) was used to identify the severity of tooth wear, and no significant differences were found between the groups of individuals with probable bruxism and those without probable bruxism.

However, tooth wear was mostly moderate in individuals with probable bruxism, which agrees with the findings of Bartolucci *et al*., who used the same assessment system and reported that the sleep bruxism group experienced tooth wear with dentine exposure, with no significant differences (28). One explanation could be the etiology of tooth surface loss which is a combination of normal physiological functional and pathological wear. Also, this pathology has multifactorial etiology and can be associated with dental erosion from dietary or gastric sources, abrasion and abfraction (27).

In this study, no significant differences were found between variables associated with probable bruxism such as sex, age, education level, and type of insurance. These results are similar to those of Yildirim *et al*., who evaluated the effects of bruxism in an adult population and reported no associations with demographic characteristic such as sex, age and level of education (13). However, most participants in that study had a higher level of education, contrary to our study where the majority had a high level of technical education. This could be explained due to the differences between economic level and social condition in participants from these studies.

Other studies have identified other stress factors, such as job characteristics, lifestyle factors, negative coping strategies and negative personality traits (29). It must be highlighted that stress risk factors may also be present in retired police officers and family members. People under stress are highly likely to develop bruxism (30), which can have an impact on their oral health and affect their well-being or quality of life.

This was a cross-sectional study; hence, the limitations involve inability to determine causal relationships between the variables, and the convenience sample does not allow extrapolate the results to the population of police officers and their relatives. For this reason, further studies are needed to assess the impact of diverse types of bruxism on the OHRQoL of police officers and their family.

To the best of our knowledge, this is the first study that assessed the impact of probable bruxism on OHRQoL in police officers and their relatives. Therefore, our results highlight the importance of mental health care in police officers since diverse traumatic incidents could develop emotional stress which could affect negatively their daily activities, OHRQoL and their families.

## Conclusions

Probable bruxism had a negative effect on the oral health-related quality of life of Peruvian police officers and their relatives. Likewise, this pathology increases physical pain, psychological discomfort, social disability and handicap. Therefore, this study is relevant because this pathology has an impact on the daily activities of individuals, affecting their well-being.

## Figures and Tables

**Table 1:** Sociodemographic and clinical characteristics of the study population (n=243).

Characteristics	n (%)
Age	54.9 ± 11.8
Age of range	
18 – 35	26 (10.7)
36 – 59	114 (46.9)
60 to more	103 (42.4)
Sex	
Female	101 (41.6)
Male	142 (58.4)
Type of insured	
Active police officer	89 (36.6)
Retired police officer	85 (35.0)
Family police officer	69 (28.4)
Education level	
Primary	1 (0.4)
Secondary	61 (25.1)
Technician	114 (46.9)
Undergraduate	67 (27.6)
Score OHIP-14	13.4 ± 8.0
Probable Bruxism	
Absence	125(51.4)
Presence	118(48.6)
Tooth wear	
No tooth wear	29 (11.9)
Slight	50 (20.6)
Moderate	111 (45.7)
Severe	40 (16.5)
Extreme	13 (5.4)

Mean ± SD

**Table 2:** Bivariate analysis between factors associated with probable bruxism (n=243).

Variable	Without Probable Bruxism	With Probable Bruxism	p
	n(%)	n(%)	
Sex			0.303^a^
Female	48 (38.4)	53 (44.9)	
Male	77 (61.6)	65 (55.1)	
Age (years)	55.2 ± 11.5	54.6 ±12.1	0.692^b^
Range of years			0.280^a^
18 – 35	13 (10.4)	13 (11.0)	
36 – 59	53 (42.4)	61 (51.7)	
60 to more	59 (47.2)	44 (37.3)	
Type of insured			0.441^a^
Active police officer	48 (38.4)	41 (34.8)	
Retired police officer	46 (36.8)	39 (33.0)	
Family	31 (24.8)	38 (32.2)	
Education level			0.134^c^
Primary	1 (0.8)	0 (0.0)	
Secondary	27 (21.6)	34 (28.8)	
Technician	56 (44.8)	58 (49.2)	
Undergraduate	41 (32.8)	26 (22.0)	
Tooth wear			0.484^c^
No wear	19 (15.2)	10 (8.4)	
Slight	25 (20.0)	25 (21.2)	
Moderate	53 (42.4)	58 (49.2)	
Severe	20 (16.0)	20 (17.0)	
Extreme	8 (6.4)	5 (4.2)	
Score OHIP-14	12±7.4	14.9±8.0	0.010^b^

Mean ± Standard Deviation; a: Chi-square test; b: U- Mann‒Whitney test; c: Fisher test.

**Table 3:** Relationship between probable bruxism and oral health-related quality of life through OHIP-14.

Domains- OHIP 14	Without Probable Bruxism	With Probable Bruxism	p
	Mean ± SD	Mean ± SD	
Functional limitation	1.3 ± 1.5	1.6 ± 1.7	0.134
Physical pain	1.5 ± 1.4	2.1 ± 1.6	0.001
Psychological discomfort	0.8 ± 1.2	1.3 ± 3.1	0.012
Physical disability	2.9 ± 1.5	2.9 ± 1.6	0.848
Psychological disability	3.2 ± 1.7	3.6 ±1.7	0.079
Social disability	1.4 ± 1.6	1.9 ± 1.5	0.002
Handicap	1.0 ± 1.5	1.4 ± 1.4	0.007
Score OHIP-14	23.0 ± 13.7	28.3 ± 15.9	0.012

U-Man Whitney test.

**Table 4:** Multivariate analysis between OHRQoL and Probable Bruxism adjusted for confounding variables.

Variables	Unadjusted			Adjusted		
	OHIP-14			OHIP-14		
	Coefficient β	IC 95%	p	Coefficient β	CI 95%	P
Probable Bruxism						
Absent	Ref.			Ref.		
Presence	2.86	0.86 - 4.87	0.005	2.38	0.37 - 4.39	0.021
Age (years)	0.08	-0.10 - 0.16	0.085	0.80	-0.11 - 0.27	0.153
Sex						
Female	Ref.			Ref.		
Male	-1.04	-3.10 - 1.02	0.320	0.36	-2.15 - 2.88	0.777
Type of insured						
Active police officer	Ref.			Ref.		
Retired police officer	0.24	-2.13 - 2.61	0.842	-1.91	-5.04 - 1.22	0.230
Family	3.38	0.87 - 5.88	0.008	1.35	-2.20 - 4.90	0.454
Education level						
Primary	Ref.			Ref.		
Secondary	-7.03	-22.69 - 8.62	0.377	-7.19	-23.34 - 8.95	0.381
Technical	-9.89	-25.49 - 5.70	0.213	-8.72	-25.04 - 7.60	0.293
Undergraduate	-11.61	-27.26 - 4.03	0.145	-10.03	-26.32 - 6.27	0.227
Tooth wear						
No wear	Ref.			Ref.		
Slight	2.45	-1.24 - 6.14	0.191	1.88	-1.75 - 5.52	0.309
Moderate	3.56	0.26 - 6.85	0.035	3.09	-0.19 - 6.37	0.065
Severe	3.11	-0.74 - 6.97	0.113	2.51	-1.39 - 6.42	0.206
Extreme	3.03	-2.25 - 8.31	0.259	2.16	-3.29 - 7.61	0.436

CI: Confidence Interval; Linear multivariate model; adjusted for sex, age, type of insured, education level and type of tooth wear.

## Data Availability

The datasets used and/or analyzed during the current study are available from the corresponding author.
